# The different expression of TRPM7 and MagT1 impacts on the proliferation of colon carcinoma cells sensitive or resistant to doxorubicin

**DOI:** 10.1038/srep40538

**Published:** 2017-01-17

**Authors:** Alessandra Cazzaniga, Claudia Moscheni, Valentina Trapani, Federica I. Wolf, Giovanna Farruggia, Azzurra Sargenti, Stefano Iotti, Jeanette A. M. Maier, Sara Castiglioni

**Affiliations:** 1 Dipartimento di Scienze Biomediche e Cliniche L. Sacco, Università di Milano, Via G.B. Grassi 74, Milano I-20157, Italy; 2 Istituto di Patologia Generale, Facoltà di Medicina, Università Cattolica del Sacro Cuore, Largo F. Vito 1, Roma I-00168, Italy; 3 Dipartimento di Farmacia e Biotecnologie, Università Alma Mater di Bologna, Via San Donato 19/2, Bologna I-40127, Italy; 4 Istituto Nazionale Biostrutture e Biosistemi, Viale delle Medaglie d’oro 305, Roma I-00136, Italy

## Abstract

The processes leading to anticancer drug resistance are not completely unraveled. To get insights into the underlying mechanisms, we compared colon carcinoma cells sensitive to doxorubicin with their resistant counterpart. We found that resistant cells are growth retarded, and show staminal and ultrastructural features profoundly different from sensitive cells. The resistant phenotype is accompanied by the upregulation of the magnesium transporter MagT1 and the downregulation of the ion channel kinase TRPM7. We demonstrate that the different amounts of TRPM7 and MagT1 account for the different proliferation rate of sensitive and resistant colon carcinoma cells. It remains to be verified whether they are also involved in the control of other “staminal” traits.

TRPM7, which belongs to the transient receptor potential melastatin subfamily, is a bifunctional protein comprising an ion channel region covalently linked to a serine/threonine α-kinase domain^1^,^2^. TRPM7, which is ubiquitously expressed, conducts several divalent cations^3^ and contributes to intracellular magnesium (Mg) homeostasis^4^, while the α-kinase phosphorylates annexin A1, myosin II, eEF2 and PLCγ2^5^,^6^,^7^,^8^. It was shown that the TRPM7 kinase domain can be proteolytically cleaved and then transported to the nucleus where it phosphorylates histones thus contributing to remodeling chromatin^9^. Knock-out studies in mice and zebrafish demonstrated that *TRPM7* is necessary for early embryonic development^4^,^10^,^11^,^12^. Silencing *TRPM7* in cultured cells disclosed its implication in the regulation of crucial cellular processes, among which cell adhesion, survival, migration, differentiation and proliferation^1^. *In vitro*, silencing *TRPM7* inhibits cell growth and impairs cell viability of all cell types tested^1^, apart from human macrovascular endothelial cells^13^,^14^. Recently, TRPM7 has emerged as a potential player in cancer^15^. *TRPM7* over-expression has been reported in human breast and pancreatic cancer and correlates with clinico-pathologic parameters^15^. Moreover, TRPM7 is required for proliferation and migration of several types of tumor cells^15^. It is noteworthy that the growth of *TRPM7* deficient cells can be in part rescued by the overexpression of another ubiquitously expressed transporter, i.e. Mg transporter 1 (MagT1)^16^. MagT1 is a plasma membrane transporter highly selective for Mg^17^. Its upregulation is described in hepatomas^18^ while its low expression correlates with a poor outcome in patients with ovarian carcinoma^19^. In T-lymphocytes MagT1 mediates a rapid Mg influx after receptor stimulation, thus indicating that MagT1 is implicated in immune response^16^,^20^,^21^.

Since very little is known about these transporters in drug resistance, we focused on TRPM7 and MagT1 expression in LoVo cells sensitive (LoVo-S) or resistant (LoVo-R) to doxorubicin (DXR). In a recent study, we have demonstrated lower amounts of TRPM7 in LoVo-R than in LoVo-S^22^. Interestingly, silencing *TRPM7* protects LoVo-S from the toxic effects of DXR, while calpeptin-induced upregulation of TRPM7 impairs the resistance of LoVo-R to the drug, thus indicating that TRPM7 might play a role in chemoresistance^22^. Here we broaden our previous studies by comparing the morphology, proliferation, expression and role of TRPM7 and MagT1 in LoVo-R and LoVo-S.

## Results

### LoVo-R vs LoVo-S: morphology and growth rate

**Figure 1:**
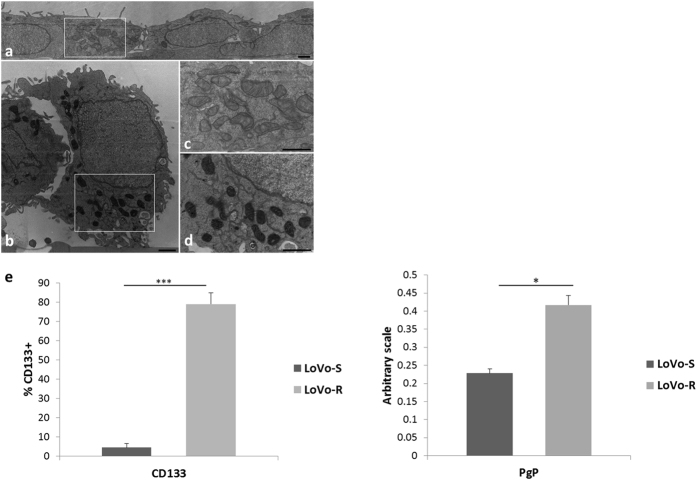
Ultrastructural features and biochemical markers of LoVo-S and LoVo-R cells. LoVo-S (**a**) and -R (**b**) cultured in a 2D-monolayer were analyzed by TEM. LoVo-S (**c**) and -R (**d**) were analyzed at a higher magnification. Scale bar = 1 μm. (**e**) The levels of CD133 and ABCB1/P-glycoprotein were analysed by cytofluorimetry and western blot, respectively. For full-length blot and FACS profile, see Supplementary 1E.

Because cell shape is related to cellular function^23^, initially we studied the morphology of LoVo-S and LoVo-R. Supplementary Figure S1A shows the morphology of LoVo-S and -R by optical microscopy. By electron microscopy (Fig. 1), both LoVo-S and LoVo-R exhibited a high nucleocytoplasmic ratio, a well-preserved ultrastructure and a nucleus with dispersed chromatin.

LoVo-S showed an elongated flattened morphology, appeared partially overlapped, connected by junctions in some cases, and arranged with the basolateral membrane adhering to the plastic dish. The cells seem to be polarized and some microvilli can be observed on the luminal surface of the cells (Fig. 1a). On the contrary, LoVo-R exhibited a round shape, were loosely tethered to the substrate, presented surface plasma membrane protrusions distributed over the entire surface, and released many extracellular vesicles (Fig. 1b). Ultrastructural analysis also highlighted differences in the mitochondria, which were more condensed and sometimes with dilated cristae in LoVo-R (Fig. 1d) vs LoVo-S (Fig. 1c).

We also evaluated the levels of CD133, a colon cancer stem cell marker that tends to accumulate in cell protrusions, and ABCB1/P-glycoprotein (PgP), which pumps out various anticancer drugs including DXR. Figure 1e shows that CD133 and PgP were overexpressed in LoVo-R, as detected by cytofluorimetry and western blot, respectively (Supplementary S1E).

**Figure 2:**
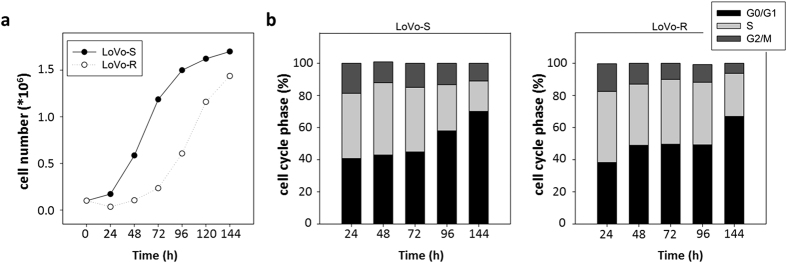
Proliferative behaviour of LoVo-S and LoVo-R cells. LoVo-S and LoVo-R cells were counted every 24 h for 6 days. Data refer to three separate experiments in triplicate ± standard deviation. (**a**) LoVo-S and LoVo-R cells were harvested at different times after seeding and cell cycle distribution was analyzed by FACS.

When we compared LoVo-R and -S for their proliferative behaviour, LoVo-R resulted growth retarded (Fig. 2a). In particular, we noticed a long lag phase in LoVo-R, which entered in the exponential phase after 48 h, while LoVo-S started to double after 24 h from seeding. LoVo-S reached the plateau phase between 72 and 96 h, when LoVo-R were still in the exponential phase. These results were confirmed when we evaluated cell cycle distribution by fluorescence-activated cell sorting (FACS) (Fig. 2b). Exponential growth occurred between 24 and 72 h in LoVo-S, and between 72 and 144 h in LoVo-R. The percentage of LoVo-S in the S phase started to decrease at 96 h, while the S phase of LoVo-R remained constant being still maximal after 96 h and tended to decrease only at 144 h (Fig. 2b).

### LoVo-R vs LoVo-S: expression and role of TRPM7 and MagT1

**Figure 3:**
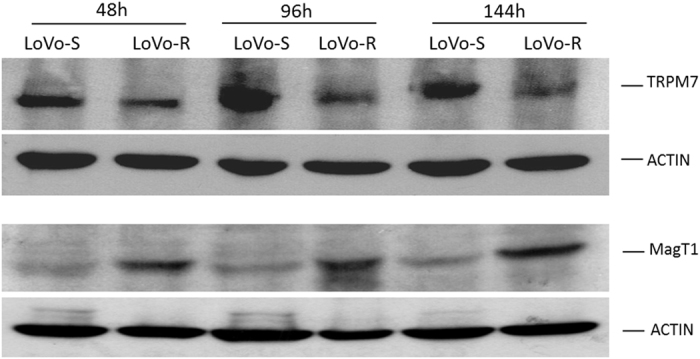
TRPM7 and MagT1 in LoVo-S and LoVo-R. LoVo cells were harvested every 48 h for 6 days and lysed. Part of the sample (100 μg) was run on a 10% SDS-PAGE and probed with anti-MagT1 antibodies. The same amounts of cell lysates were separated on a 8% SDS-PAGE and utilized for Western blot using anti-TRPM7 antibodies. Blots were processed in parallel. Actin was used as a control of loading. Full-length blots and quantification are presented in Supplementary Figure S3.

At different time points during the experiment in Fig. 2, we evaluated the levels of TRPM7 and MagT1 by western blot. We found that TRPM7 is lower while MagT1 is higher in LoVo-R than in LoVo-S at all the time points tested (Figs 3 and Supplementary S3). Moreover, by immunofluorescence, TRPM7 has a similar localization in LoVo-R and -S with a clear nuclear exclusion (Supplementary S3).

**Figure 4:**
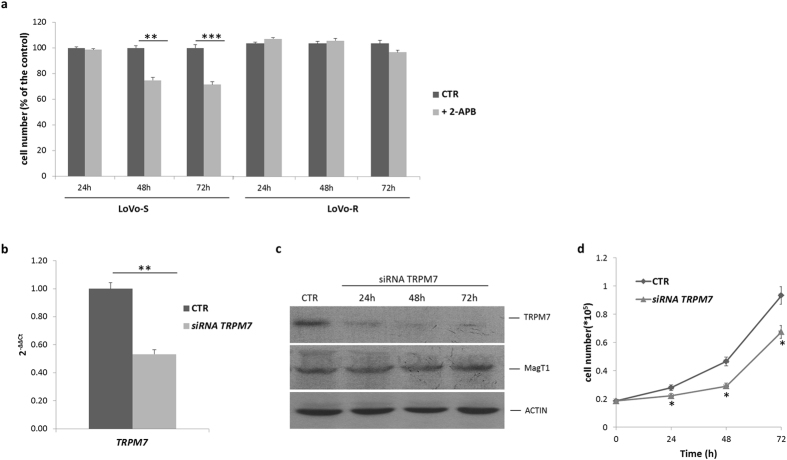
Effect of *TRPM7* silencing on LoVo-S proliferation. (**a**) Cells were treated with the TRPM7 channel blocker 2-APB (50 μM) and counted every 24 h for 3 days. Data are express as % of the control and refer to three separate experiments in triplicate ± standard deviation. (**b**) LoVo-S were transfected with a siRNA against *TRPM7* or with a non-silencing siRNA. After 24 h RNA was extracted and Real-Time PCR performed using primers designed on *TRPM7* sequence. (**c**) Western blot was performed on cell extracts of LoVo-S 24, 48 and 72 h after exposure to siRNA. Actin was used as a control of loading. Full-length blots and quantification are presented in Supplementary Figure S4C. (**d**) LoVo-S treated as described above were counted every 24 h. Data are expressed as total cell number and refer to three separate experiments in triplicate ± standard deviation.

**Figure 5:**
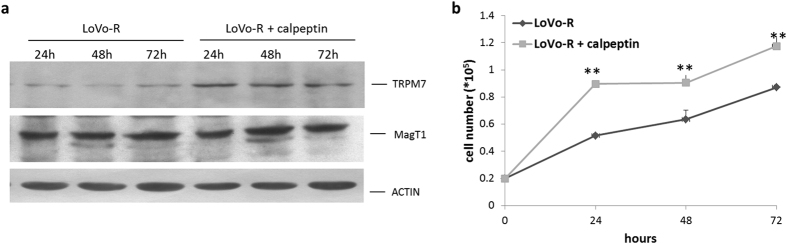
Effects of calpeptin on LoVo-R proliferation. LoVo-R cells were treated with calpeptin (5.0 μg/ml) for 24, 48 and 72 h. (**a**) Western blot was performed on cell lysates with anti-TRPM7 and anti-MagT1 antibodies. Actin was used to show that equal amounts of proteins were loaded per lane. Full-length blots and quantification are presented in Supplementary Figure S5A. (**b**) LoVo-R were counted every 24 h. Data are expressed as total cell number and refer to three separate experiments in triplicate ± standard deviation.

Because TRPM7 has been linked to the control of cell proliferation^1^, we determined whether TRPM7 might participate to the regulation of LoVo cell growth. Initially, we treated LoVo-S and -R with 2-aminoethoxydiphenyl borate (2-APB, 50 μM), a commonly used non-specific blocker of TRPM7 activity^22^. While no effect was observed in LoVo-R upon exposure to the compound, we found that 2-APB hindered LoVo-S cell growth (Fig. 4a). Then we transiently transfected LoVo-S with a specific siRNA for *TRPM7* or with a non-silencing, scrambled siRNA as a control. After 24 h, Real-Time PCR showed a marked reduction of *TRPM7* transcript (Fig. 4b). By western blot we detected dramatically reduced amounts of TRPM7 up to 72 h after silencing and no modulation of MagT1 (Figs 4c and Supplementary S4C). TRPM7 downregulation correlated with the reduction of LoVo-S cell growth (Fig. 4d). The same siRNA exerted no significant effects on the growth of LoVo-R (Supplementary S4D). The role of TRPM7 in modulating LoVo cell proliferation is supported also by experiments on LoVo-R cultured in the presence of the calpain inhibitor calpeptin. The concentration of calpeptin (5 μg/ml) used in this study exerts no cytotoxic effect^22^. Figure 5a and b show that calpeptin upregulated TRPM7, but not MagT1 (Supplementary S5A), and induced cell proliferation.

**Figure 6:**
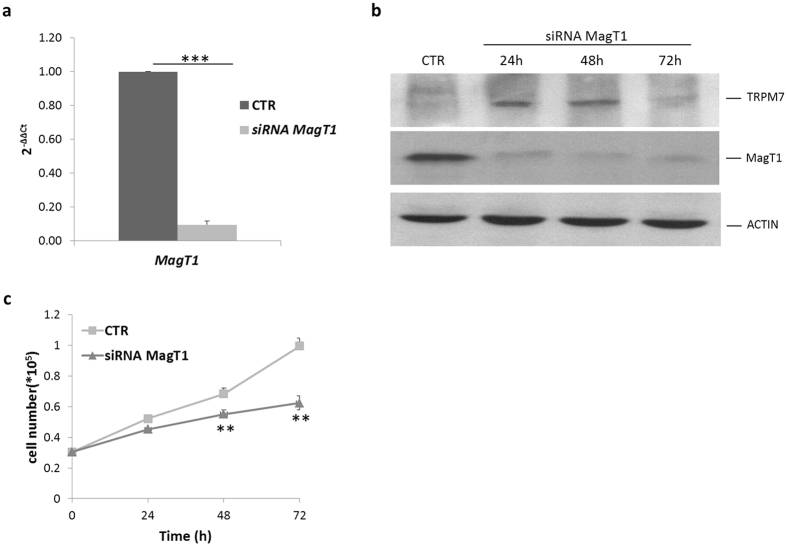
*MagT1* silencing and LoVo-R proliferation. LoVo-R were transfected with a siRNA against *MagT1* or with a non-silencing siRNA. (**a**) After 24 h Real-Time PCR was performed using primers designed on *MagT1* sequence. (**b**) Western blot was performed on cell extracts of LoVo-R 24, 48 and 72 h after transfection with siRNA against *MagT1*. Actin was used as a control of loading. Full-length blots and quantification are presented in Supplementary Figure S6B. (**c**) LoVo-R were counted every 24 h. Data are expressed as total cell number and refer to three separate experiments in triplicate ± standard deviation.

Then, we transiently silenced *MagT1* in LoVo-R. After 24 h Real-Time PCR demonstrated the downregulation of *MagT1* transcript (Fig. 6a). At various time points after silencing, western blot showed the reduction of the total amounts of MagT1 (Figs 6b and Supplementary S6B). It is noteworthy that *TRPM7* is upregulated early and transiently during *MagT1* silencing. However, cell proliferation was severely impaired in *MagT1*-deficient LoVo-R (Fig. 6c).

## Discussion

We have previously demonstrated that TRPM7 expression is lower in confluent LoVo-R than in confluent LoVo-S, and contributes to render the cells resistant to DXR^22^. Here we show that the resistant phenotype is characterized not only by low amounts of TRPM7 but also by high levels of MagT1. These differences are maintained in sparse and in exponentially growing LoVo cells and are independent from their cell cycle position. It is interesting to recall that similar results were obtained in human endothelial cells from the umbilical vein for TRPM7^13^ and MagT1 (unpublished results).

Several studies have shown that TRPM7 is required for the proliferation of normal and tumor cells^1^,^15^. Our results are in accordance with these findings since we show that (i) LoVo-R, which express low amounts of TRPM7, grow slower than LoVo-S; (ii) calpeptin-induced TRMP7 accelerates LoVo-R proliferation; and (iii) silencing *TRPM7* hinders LoVo-S cell proliferation. We rule out that TRPM7 contributes to the regulation of cell growth by impacting on intracellular Mg since we have shown an inverse correlation between total intracellular magnesium and the levels of TRPM7^22^. Since TRPM7 phosphorylates some proteins, we argue that silencing TRPM7 might impair the phosphorylation of its substrates. Among the phosphorylation targets of TRPM7, annexin 1 and phospholipase Cγ^5^,^8^ are particularly challenging, because they are involved in the regulation of cell growth.

Far less is known about the involvement of MagT1 in cell proliferation. It is reported that the overexpression of MagT1 in TRPM7-deficient DT40 cells restores cell growth^16^. We found a marked inhibition of the proliferation of LoVo-R silencing *MagT1* in spite of a transient induction of the levels of TRPM7. We hypothesize that such an upregulation does not reach the threshold necessary to sustain cell growth. It is noteworthy that silencing *MagT1* did not alter total intracellular Mg (Supplementary S6D). Moreover, since MagT1 is a subunit of the oligosaccharyltransferase complex and, therefore, is implicated in protein glycosylation^24^, it is possible that its silencing impacts on post-translational modification of proteins involved in controlling cell proliferation.

It is also interesting to point that LoVo-R and not LoVo-S express the stem cell biomarker CD133, which is considered a putative marker for colorectal cancer stem cells^25^. This indicates that LoVo-R display a higher stemness potential than LoVo-S, as suggested also by the marked differences of cell shape and by our previous results^22^. To this purpose, it should be highlighted that i) most primitive stem cells seem to grow slowly, and ii) they express detoxification molecules, i.e ABC transporters^26^. Indeed, LoVo-R grow slower and express higher amounts of PgP, which mediates DXR efflux^26^, than LoVo-S. Another issue to consider is that CD133 is selectively localized in microvilli and other plasma membrane protrusions^27^ and electron microscopy clearly shows that LoVo-R are richer in plasma membrane protrusions than LoVo-S. Electron microscopy also shows that the mitochondria in LoVo-R, while retaining a normal ultrastructure, have a dense matrix. It has been reported that cells with dense mitochondria are hypoxia-tolerant, and, consequently, generate adequate amounts of ATP by oxidative phosphorylation^28^, a feature which is fundamental to boost the production of ATP necessary to extrude DXR via PgP and to cope with constant chemotherapeutic stress^29^. Moreover, an enhanced oxidative metabolism in drug-resistant vs drug-sensitive cells was demonstrated in glioma^30^. Accordingly, the metabolism of colon cancer cells shifts from glycolysis towards oxidative phosphorylation upon exposure to anticancer drugs^31^.

In conclusion, we demonstrate that resistant cells are more staminal than sensitive cells and this phenotype associates with different amounts of MagT1 and TRPM7, which are, in part, responsible for the different proliferation of LoVo-R and LoVo-S.

## Methods

### Cell Culture

LoVo-S and LoVo-R (donated by Dr. P. Perego, Istituto Nazionale Tumori, Milano) were cultured in DMEM containing 10% fetal bovine serum and 2 mM glutamine, at 37 °C and 5% CO_2_^22^.

To obtain a transient downregulation of *MagT1* and *TRPM7*, we utilized the stealth siRNAs developed by Qiagen for TRPM7^22^ and Invitrogen for MagT1. siRNAs targeting *TRPM7* were transfected using HiPerFect Transfection Reagent (Qiagen) while for siRNAs targeting *MagT1* we used Lipofectamine RNAiMAX (Invitrogen). Non-silencing, scrambled sequences were used as controls (CTR).

For proliferation assays, the cells were seeded at low density (10000 cells/cm^2^), trypsinized, stained with a trypan blue solution (0.4%) and counted using a cell counter (Logos Biosystems).

Cell cycle was analyzed by FACS. Briefly, 1 × 10^6^ cells were centrifuged and resuspended in a hypotonic staining solution (Na_3_citrate 1 mg/mL, Nonidet 0.1%, Rnase 1 μg/mL, Propidium Iodide 50 μg/mL) and left overnight at 4 °C. Cells were analyzed on a Brite HS BioRad flow cytometer, and the PI fluorescence was excited at 360 nm and collected at 600 nM on a linear scale. The percentages of the cells in the different phases of the cycle were evaluated by using the Modifit software (Verity).

CD133 expression was evaluated by flow cytometry, using the AC133 mouse monoclonal antibody directly conjugated with phycoerythrin (Miltenyi Biotech).

### Transmission Electron Microscopy (TEM)

The semi-confluent cells were fixed overnight at 4 °C in a solution containing 2.5% glutaraldehyde in 0.1 M cacodylate buffer (pH 7.4), immobilized on a Nunc Sylgard coated Petri dish (ThermoFisher Scientific), rinsed twice in the same buffer for 20 min and processed for TEM. After post-fixation with 1% osmium tetroxide at 0 °C for 30 min, the 2D-monolayer cultures *in situ* on Petri were washed in distilled water, stained with 2% aqueous uranyl acetate, dehydrated in graded ethanols at 4 °C and embedded in Epon-Araldite resin. Ultra-thin sections cut by a Leica Supernova ultramicrotome (Reichert Ultracut E and UC7; Leica Microsystems) were stained with lead citrate and observed under a Zeiss EM10 electron microscope.

### Real-Time PCR

Total RNA was extracted by the PureLink RNA Mini kit (Ambion). Single-stranded cDNA was synthesized using High Capacity cDNA Reverse Transcription Kit (Applied Biosystems) and Real-time PCR was performed using TaqMan Gene Expression Assays (Life Technologies): Hs00918928_g1 (*TRPM7*) and *MagT1* (Hs00997540_m1). The housekeeping gene *GAPDH* (Hs99999905_m1) was used as an internal reference gene. Relative changes in gene expression (defined as FC) were analyzed by the 2^−ΔΔ*Ct*^ method^32^. Experiments were performed twice in triplicate.

### Western Blot Analysis

Cells were lysed in 50 mM TrisHCl pH 8.0 containing 150 mM NaCl, 1 mM EDTA, 1% NP-40. Cell extracts (100 μg/lane) were resolved by 8 or 10% SDS-PAGE, transferred to nitrocellulose sheets at 100 mA for 16 h, and probed with anti-TRPM7 (Bethyl), anti-MagT1 (ProteinTech), anti-PgP (ThermoScientific), and anti-actin (Sigma Aldrich) antibodies. Secondary antibodies were labelled with horseradish peroxidase (GE Healthcare). The SuperSignal chemiluminescence kit (Pierce) was used to detect immunoreactive proteins. All the results were reproduced at least three times and a representative Western blot is shown. Densitometric analysis was performed by the ImageJ software and ratio between the protein of interest and actin was calculated on three separate experiments (see Supplementary).

### Statistical Analysis

Statistical significance was determined using Student’s t test and set as following: *P < 0.05, **P < 0.01, ***P < 0.001.

## Additional Information

**How to cite this article:** Cazzaniga, A. *et al*. The different expression of TRPM7 and MagT1 impacts on the proliferation of colon carcinoma cells sensitive or resistant to doxorubicin. *Sci. Rep.* **7**, 40538; doi: 10.1038/srep40538 (2017).

**Publisher's note:** Springer Nature remains neutral with regard to jurisdictional claims in published maps and institutional affiliations.

## Supplementary Material

Supplementary Information
