# Supplementary material for: The different expression of TRPM7 and MagT1 impacts on the proliferation of colon carcinoma cells sensitive or resistant to doxorubicin
Source: Sci Rep. 2017 Jan 17;7:40538. doi: 10.1038/srep40538 (PMC5240092; doi:10.1038/srep40538)

The different expression of TRPM7 and MagT1 impacts on the proliferation of colon carcinoma cells sensitive or resistant to doxorubicin

Alessandra Cazzaniga, Claudia Moscheni, Valentina Trapani, Federica Wolf, Giovanna Farruggia, Azzurra Sargenti, Stefano Iotti, Jeanette AM Maier, Sara Castiglioni

Supplementary Figures

Figure S1A

Morphology of LoVo-S and -R.

Light microscopy (x100) of a semi-thin section showing a representative area of 2D-monolayer LoVo-S (a) and LoVo-R (b) cultures in situ on Petri stained with 0.5% toluidine blue in 1% sodium borate.

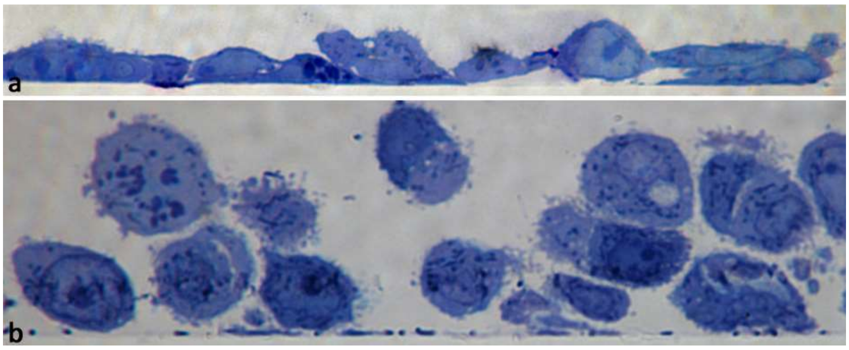

Figure S1E

Level of CD133 and PgP in LoVo-R and –S

CD133 levels were evaluated by flow cytometry, using the AC133 mouse monoclonal antibody directly conjugated with phycoerythrin (Miltenyi Biotech).

Western blot was performed using antibodies against PgP. Actin was used as a control of loading. A representative blot is shown.

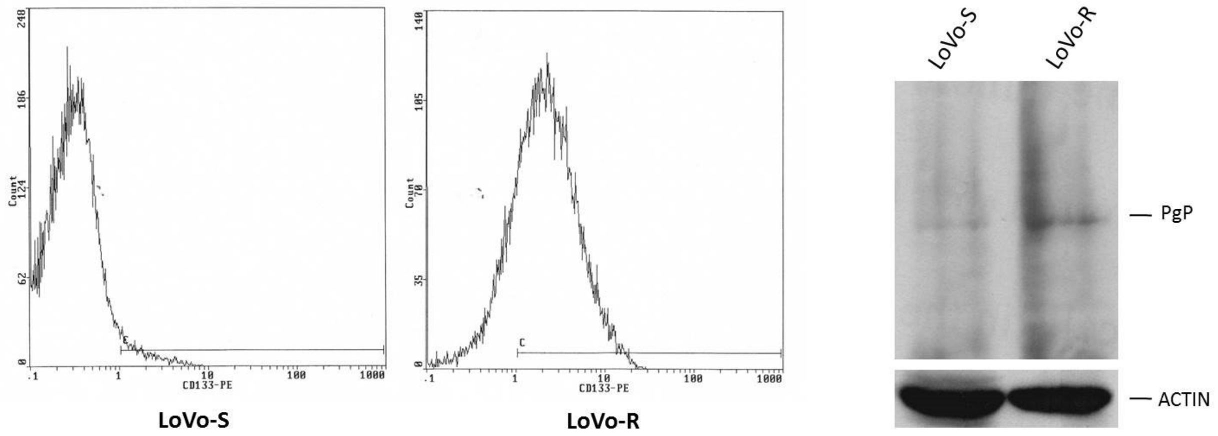

Figure S3

Levels and subcellular localization of TRPM7 and MagT1 in LoVo-S and LoVo-R

Cell lysates were analysed by western blot using antibodies against TRPM7 and MagT1. Densitometric analysis was performed by the ImageJ software and TRPM7 or MagT1 /actin ratio was calculated on three separate experiments. A representative blot is shown.

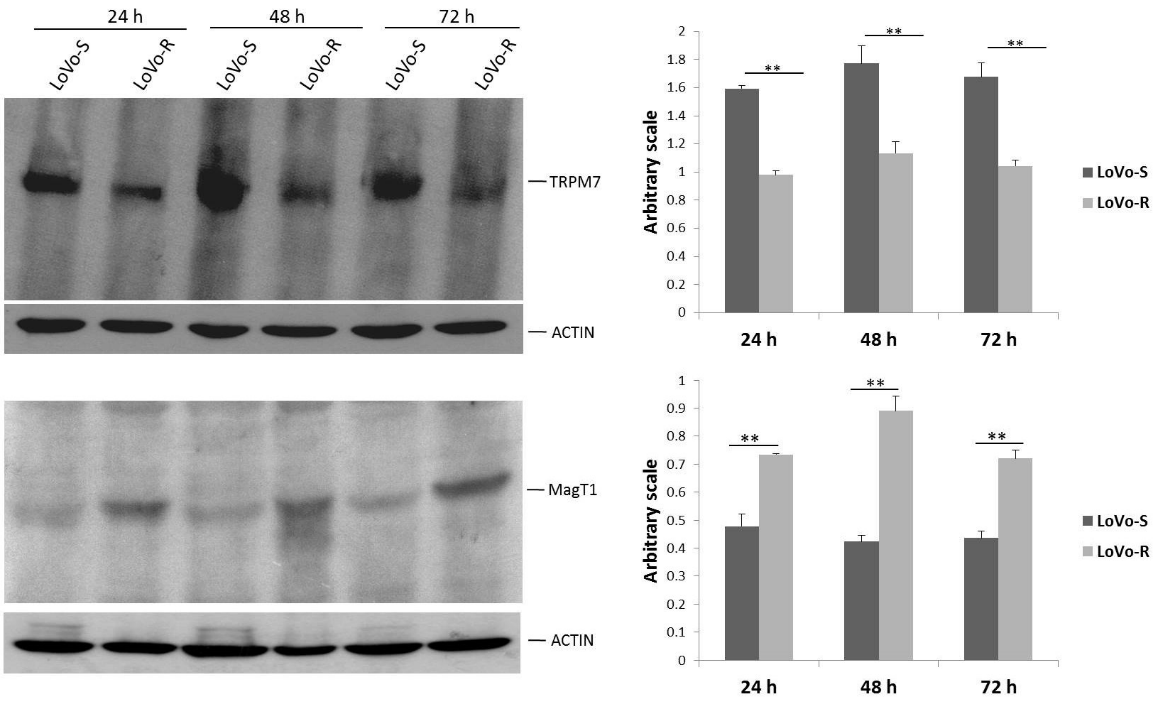

Localization of TRPM7 by immunofluorescence

Cells were grown on sterilized coverslips. Cells were then fixed in 4% paraformaldehyde, permeabilized and blocked in a solution containing 0.02% saponin, 0.2% BSA, 50 mM NH_4Cl. The primary antibody was used at a 1:100 dilution and incubated for 2 h at RT. The secondary antibody was an Alexa Fluor 546-conjugated IgG (Invitrogen), and was used at a 1:400 dilution, applied for 1 h at RT. Nuclei were counterstained with Hoechst 33258 (0.1 ug/uL). Images (512 × 512 pixels) were acquired with a confocal laser scanning system (TCS-SP2, Leica Microsystem, Wetzlar, Germany). Alexa Fluor 546 fluorescence was excited with a HeNe laser, while Hoechst staining was imaged after two-photon excitation with an ultrafast, tunable, mode-locked titanium: sapphire laser (Chamaleon, Coherent Inc., Santa Clara, CA). Scale bar 20 µm.

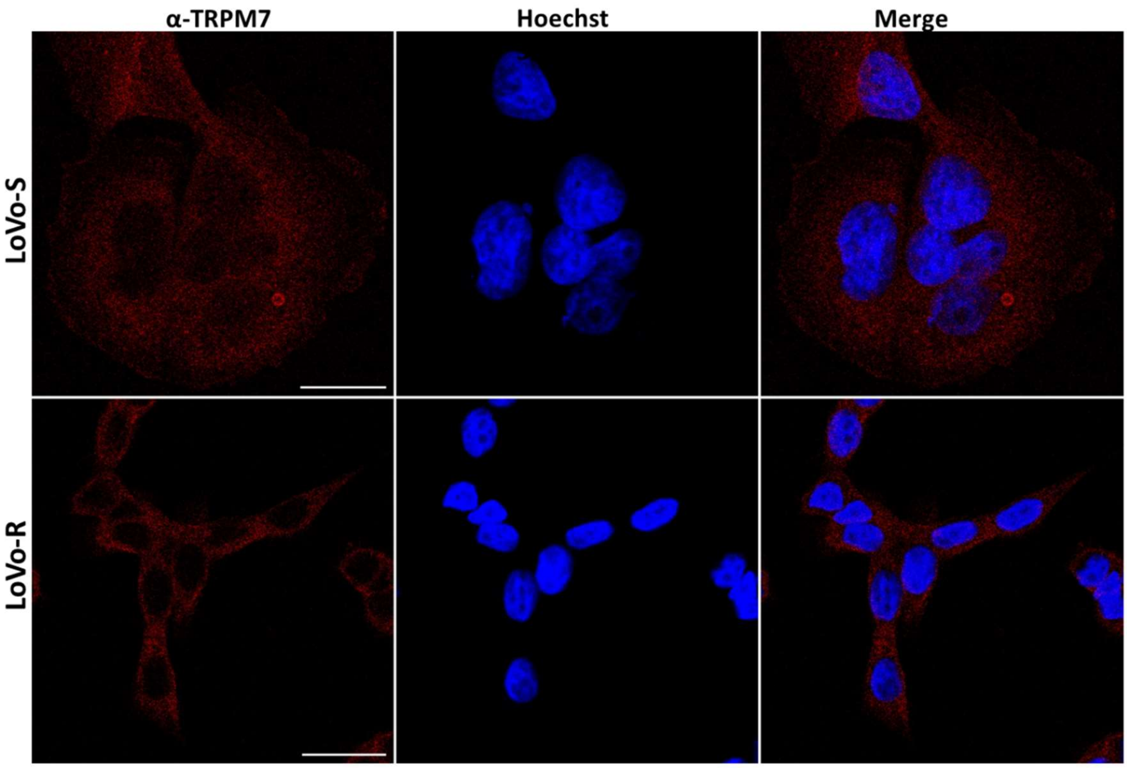

Figure S4C

Levels of TRPM7 and MagT1 after silencing TRPM7 in LoVo-S.

Western blot was performed on cell extracts of LoVo-S 24, 48 and 72 h after exposure to siRNA targeting TRPM7. Cell lysates were analysed by western blot using antibodies against TRPM7 and MagT1. Densitometric analysis was performed by the ImageJ software and TRPM7 or MagT1 /actin ratio was calculated on three separate experiments. A representative blot is shown.

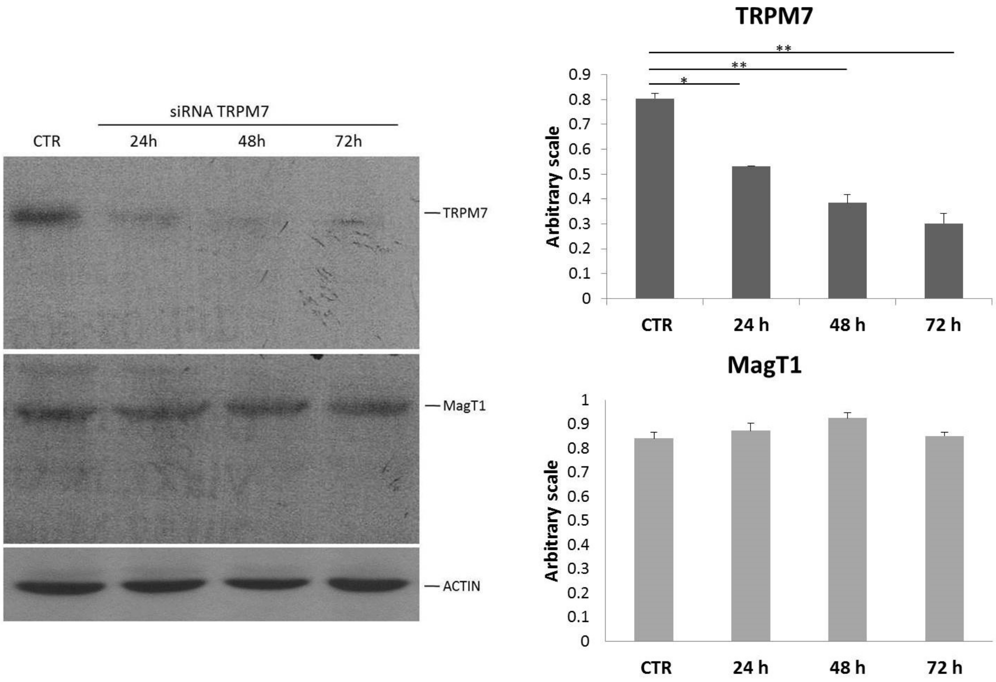

Figure S4D

LoVo cell proliferation after transfection with siRNA targeting *TRPM7*.

LoVo-R and LoVo-S were transiently transfected with a specific siRNA against *TRPM7* or with a non-silencing siRNA sequence as a control. After 72h, the cells were counted. Data are expressed as % of the control ± standard deviation.

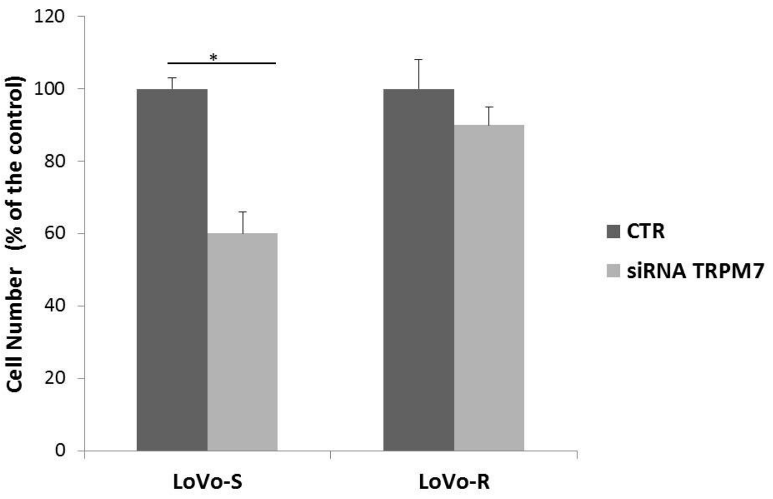

Figure S5A

Levels of TRPM7 and MagT1 after exposure to calpeptin.

LoVo-R were treated with calpeptin (5.0 µg/ml) for 24, 48 and 72 h. Western blot was performed with anti-TRPM7 and anti-MagT1 antibodies. Densitometric analysis was performed by the ImageJ software and TRPM7 or MagT1 /actin ratio was calculated on three separate experiments. A representative blot is shown.

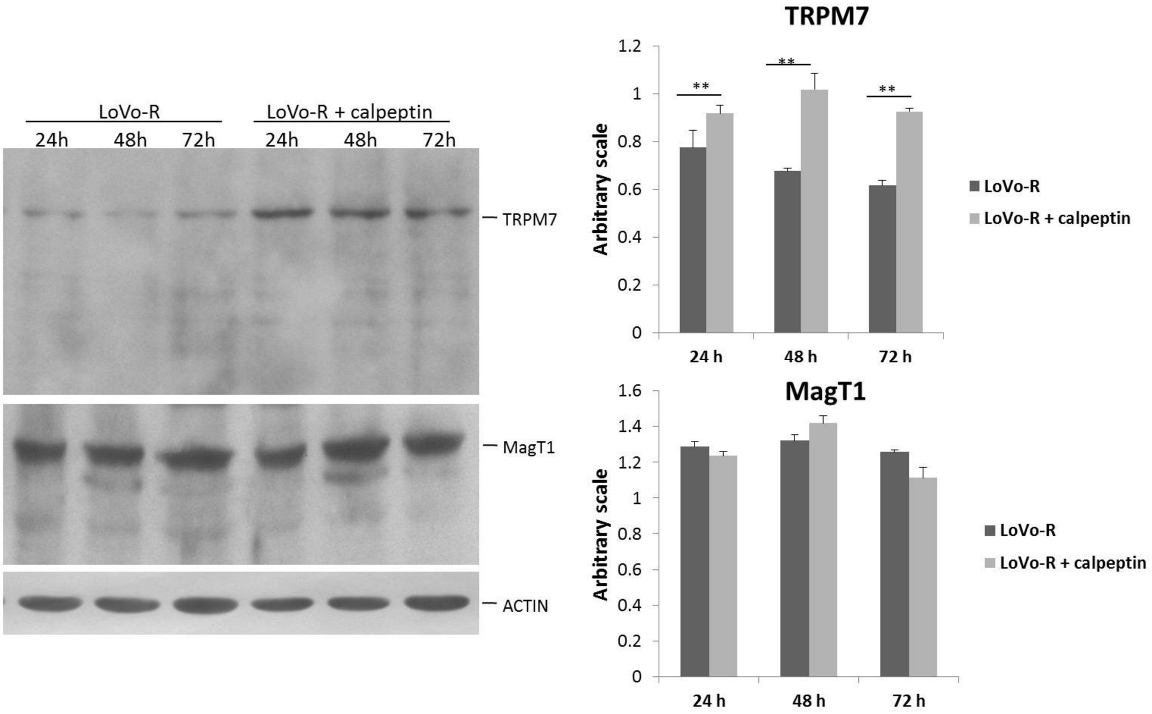

Figure S6B

Levels of TRPM7 and MagT1 after silencing MagT1 in LoVo-R.

Western blot was performed on cell extracts of LoVo-S 24, 48 and 72 h after exposure to siRNA targeting MagT1. Cell lysates were analysed by western blot using antibodies against TRPM7 and MagT1. Densitometric analysis was performed by the ImageJ software and TRPM7 or MagT1 /actin ratio was calculated on three separate experiments. A representative blot is shown.

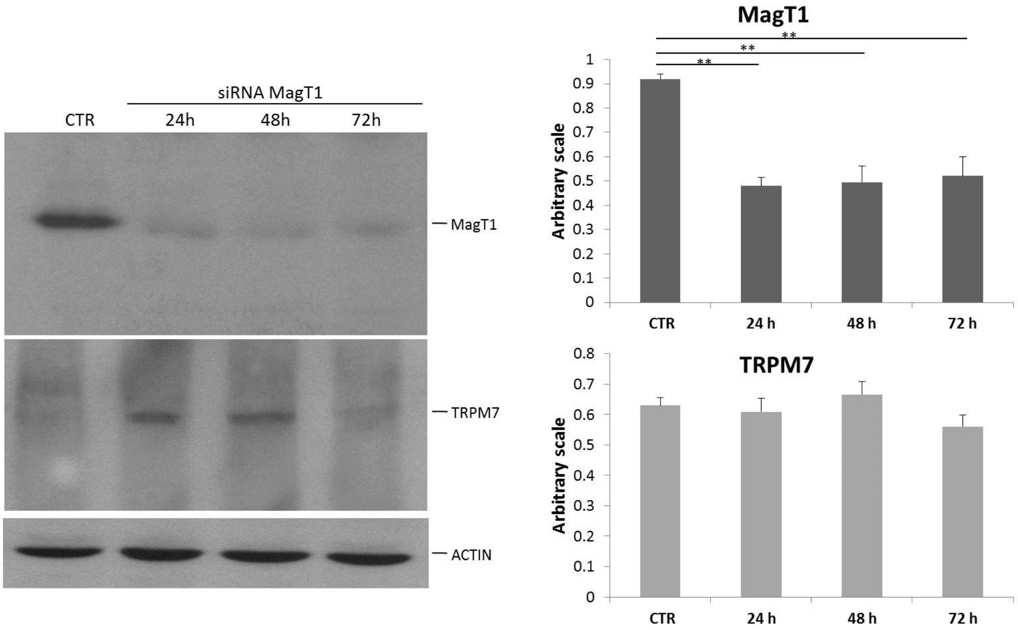

Figure S6D

Total intracellular Mg in LoVo-R after silencing Magt1.
LoVo-R were transiently transfected with siRNA against *MagT1* or with a non-silencing siRNA for 24 h. Total intracellular Mg was measured using the fluorescent chemosensor DCHQ5.

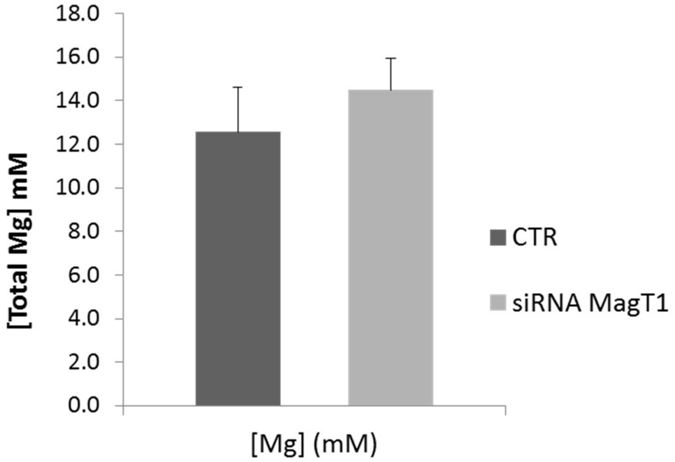

Supplement: Supplementary Information [file srep40538-s1.pdf]
